# Analysis of povidone iodine, chlorhexidine acetate and polyhexamethylene biguanide as wound disinfectants: in vitro cytotoxicity and antibacterial activity

**DOI:** 10.1136/bmjnph-2022-000431

**Published:** 2023-04-04

**Authors:** Mingshi Zhang, Jian Jin, Yingying Liu, Chi Ben, Haihang Li, Dasheng Cheng, Yu Sun, Wang Guang-Yi, Shihui Zhu

**Affiliations:** 1 Department of Burns, Wenzhou Medical University, Wenzhou, Zhejiang, China; 2 Department of Polymer Science, Fudan University, Shanghai, China; 3 Shanghai Depeac Biotechnology Co., Ltd, Shanghai, China; 4 Department of Burns, Naval Medical University, Yangpu, Shanghai, China; 5 Department of Burns, Changhai Hospital, Yangpu, Shanghai, China

**Keywords:** Infectious disease, Skin disorders

## Abstract

**Objectives:**

Even though disinfectants are commonly used in clinical practice and daily life, there are few studies on their antibacterial ability and cytotoxicity, which are closely related to the safety and effectiveness of their use. To provide a basis for the use of disinfectants, the cytotoxicity and antibacterial activity of three most commonly used disinfectants, povidone-iodine, chlorhexidine acetate and polyhexamethylene biguanide (PHMB), were investigated.

**Design:**

A CCK-8 assay was used to measure the activities of human fibroblasts (HF) and keratinocytes (HaCat), the two most important cells in wound healing, following their exposure to disinfectants. The effects of different times and concentrations were included. The antibacterial activity of disinfectants against *Staphylococcus aureus, Acinetobacter baumannii, Klebsiella pneumoniae* was reflected by their minimum inhibitory concentration and minimum bactericidal concentration.

**Results:**

All three disinfectants showed strong cytotoxicity in direct contact with HF and HaCat cells. Cytotoxicity increased with increasing exposure time and concentration. *S. aureus*, *A. baumannii and K. pneumoniae* comprised 70%, 55% and 85% of the strains sensitive to povidone iodine; 50%, 45% and 80% of the strains sensitive to chlorhexidine acetate; and 60%, 45% and 80% of the strains sensitive to PHMB, respectively.

**Conclusions:**

All three disinfectants were cytotoxic; therefore, it is necessary to pay attention to the use time and concentration in the clinical setting. All three disinfectants were cytotoxic, with povidone-iodine being the most cytotoxic even at low concentrations. PHMB had better antibacterial efficacy against *S. aureus* and is suitable for the treatment of shallow wounds primarily. All three tested bacteria were significantly more sensitive to PHMB than to the other disinfectants.

**WHAT IS ALREADY KNOWN ON THIS TOPIC**

- Disinfectants are commonly used in clinical practice and daily life, especially during wound treatment. It has certain cytotoxicity, which will affect wound healing.

**WHAT THIS STUDY ADDS**

- In this study, the bactericidal abilities and toxicities of common disinfectants were compared, especially the effect of action time and concentration on cytotoxicityty.

**HOW THIS STUDY MIGHT AFFECT RESEARCH, PRACTICE OR POLICY**

- The systematic analyses of the bactericidal ability and toxicity of common disinfectants provide a basis for the selection of disinfectants to treat wounds.

## Background

The prevention and control of infection is vital for wound treatment. The administration of a topical disinfectant is the main method used to treat infected wounds^1–3^ When a disinfectant is applied to a wound, it has a toxic effect on the cells in the wound tissue because the barrier function of the skin is absent and the disinfectant can have direct contact with the wound tissue.^4 5^ Therefore, the recommended disinfectant concentration for wound treatment is often lower than that prescribed for application to intact skin. To the best of our knowledge, few studies have explored the toxic effects of disinfectants on wound tissues. In practice, pathogenic bacteria are resistant to several disinfectants.^6 7^ As such, new disinfectants have been applied clinically to manage this problem. It is necessary to understand the efficacy of disinfectants and the relative sensitivity of wound bacteria to these agents to reduce infection resistance so that healing may be promoted in wounds.

Here, we selected three disinfectants commonly used in clinical practice and evaluated their cytotoxicity to wound cells and the influence of contact time and concentration on their cytotoxic ability. We also investigated the antibacterial efficacy and relative sensitivity of bacteria commonly found in wounds. Thus, we aimed to provide an empirical basis for the rational use of topical disinfectants in clinical practice.

## Materials and methods

Human fibroblasts (HF) (ATCC CRL-1634) and HaCat (ATCC PTA-9170) were purchased from the American Type Culture Collection (Manassas, Virginia, USA). The cultures contained 10% (v/v) fetal bovine serum and were maintained at 37°C in a 5% humidified CO_2_ atmosphere.

The disinfectants tested in this study were povidone-iodine,^8^ chlorhexidine acetate^9^ and polyhexamethylene biguanide (PHMB)^10^ (Shanghai Haijie Biotechnology, Shanghai, China). The standard concentrations recommended for wound treatment were used in the experiments: 0.5%, 0.05% and 0.1% for povidone iodine, chlorhexidine acetate and PHMB, respectively.

The bacterial strains used in this study were *Klebsiella pneumoniae* CMCC (B) 46117,^11^ *Acinetobacter baumannii* ATCC19606^12^ and *Staphylococcus aureus* ATCC6538^13^ (China General Microbiological Culture Collection Center, Beijing, China). Clinical strains were provided by Shanghai Changhai Hospital (Shanghai, China) and isolated from wounds. Twenty strains of each bacterial species were selected.

### In vitro cytotoxicity after direct contact with a disinfectant

#### Cytotoxicity after short-term exposure to standard disinfectant concentrations

This parameter indicated the direct toxic effects of disinfectants on wound cells. A 100 μL of cell suspension (5×10^4^ cells/mL) was added to each well of a 96-well plate and incubated for 24 hours. Thereafter, a 100 µL of a mixture of 0.5% povidone-iodine, 0.05% chlorhexidine acetate, 0.1% PHMB and culture medium was added to each well of the plate. After 5, 30 and 180 s, 10 µL of CCK-8 was added to each well and the cells were incubated for 1 hour. Absorbance was read at 490 nm. The absorbance of the wells containing the cells and all three disinfectants was recorded as A_n_. The absorbance of the wells containing the cells and the medium was recorded as A. The absorbance of the wells containing cell-free medium was recorded as A_0_. Cell survival rates were calculated as follows:

(1) $$Cellsurvivalrate(\%)=(A_{n}-A_{o})/(A-A_{o})\times 100$$

Cytotoxicity after prolonged direct contact at various disinfectant concentrations

This parameter simulated residual disinfectant cytotoxicity resulting from extended contact with wound cells. Cells were prepared as previously described. The dilution ratios of the standard disinfectants were 1/2, 1/4, 1/8, 1/16, 1/32, 1/64, 1/128, 1/256 and 1/512. A 100 μL of povidone-iodine, chlorhexidine acetate and PHMB at various concentrations were added together with the culture medium to each well of a 96-well plate. After 0.5, 1, 2 and 4 hours, the cells were harvested and their survival rates were calculated using equation 1.

#### Effects on apoptosis

Cells were prepared as previously described. A culture medium (2 mL) containing all three disinfectants at 1/512, 1/64 and 1/8 dilutions was added to the culture plate. Apoptosis was determined using flow cytometry. Annexin VFluorescein isothiocyanate isomer and Propidium Iodide were detected via the FITC and R-phycoerythrin channels, respectively.

### Antibacterial efficacy of disinfectant in vitro

#### Preparation of bacterial suspensions

A bacterial suspension of pneumonia CMCC (B) 46117,^12^ *A. baumannii* ATCC19606^13^ and *S.s aureus* ATCC6538^14^ at 10^8^ CFU/mL was prepared and stored at 4°C until required for subsequent testing.

#### Minimum inhibitory concentration

Bacterial suspensions (100 µL) were added to wells 1–10 of the 96-well plates. Diluted disinfectants (1/2, 1/4, 1/8, 1/16, 1/32, 1/64, 1/128, 1/256, 1/512 and 1/1024) were added to wells 1–10, 200 µL sterile Mueller Hinton broth was added to well 11 and 200 µL bacterial suspension was added to well 12. The plates were then incubated at 37°C for 22 hours. The optical density of each well was measured using a spectrophotometer. The lowest concentration of disinfectant that caused no change in turbidity was designated as the minimum inhibitory concentration (MIC) for that bacterial strain. All bacterial strains and disinfectants were assayed in triplicate.^9^

#### Minimum bactericidal concentrations

Samples obtained from the preceding MIC experiments were spread onto slides and incubated at 37°C for 7 days. The minimum concentration at which no bacterial colonies appeared was designated as the minimum bactericidal concentration (MBC). Concurrently, nutrient agar plates were inspected for the formation of colonies to determine whether bacterial contamination occurred during the experiments.^11^

#### Bacterial disinfectant susceptibility analysis

Bacterial strains with an MIC or MBC higher than that of the standard strains were considered to have reduced disinfectant susceptibility. The proportion of clinical strains with relative changes in disinfectant sensitivity was calculated.

### Statistical analysis

A t-test of independent samples was used for cytotoxicity assays and χ^2^ analysis was used for the bacterial disinfectant sensitivity change assay. A p<0.05 was considered statistically significant. All statistical analyses were performed using SPSS Statistics (V.26.0; IBM).

## Results

### In vitro cytotoxicity of direct disinfectant contact

#### Cytotoxicity after short-term exposure to standard disinfectant concentrations

**Figure 1:**
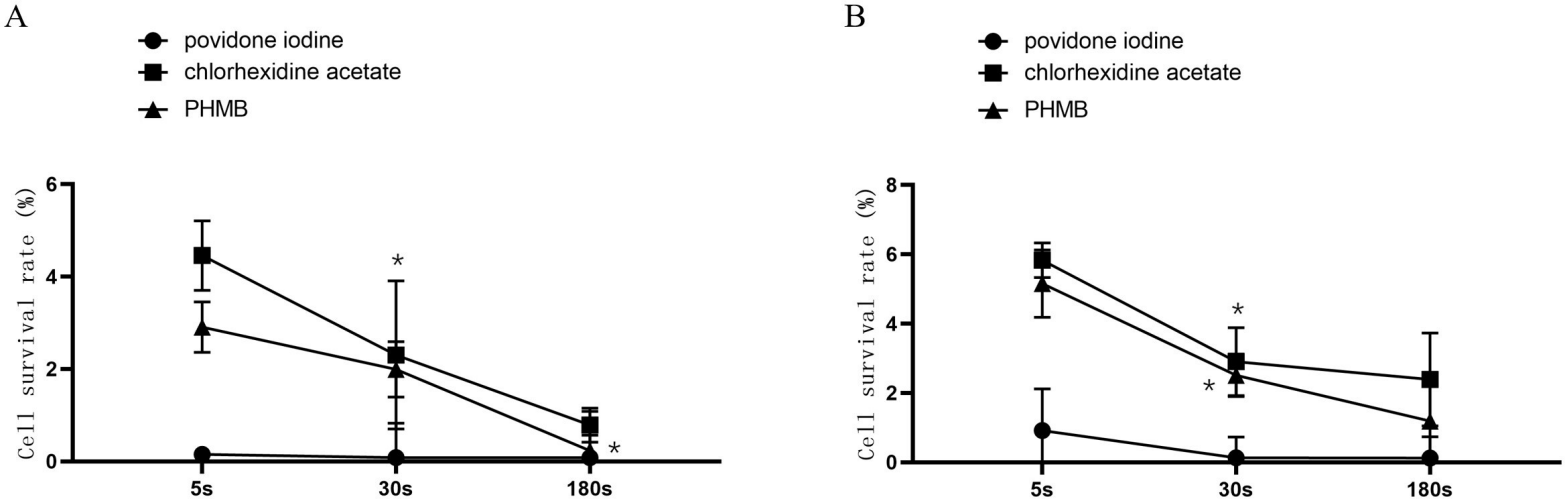
Direct contact cytotoxicity of povidone iodine, chlorhexidine acetate and PHMB at standard concentrations. *Compared with the previous time points, the difference was statistically significant (p<0.05). (A) HF. (B) HaCat. HF, human fibroblasts; PHMB, polyhexamethylene biguanide.

After relatively short cell contact times, povidone-iodine, chlorhexidine acetate and PHMB were highly cytotoxic. The HF survival rates after a contact time of 5 s with povidone iodine, chlorhexidine acetate and PHMB were 0.15%±0.13%, 4.46%±0.75% and 2.91%±0.55%, respectively. After 180 s of contact time, the HF survival rates were only 0.08%±0.49%, 0.79%±0.37% and 0.23%±0.85%, respectively. These values were significantly lower than those determined for the 5 s contact time (p<0.05). Similar results were obtained for HaCaT cells exposed to different disinfectant concentrations and contact times. The results are shown in figure 1. There was no significant difference between chlorhexidine acetate and PHMB in terms of cell survival after 5 s and 30 s exposure to these agents (p>0.05). However, the cell survival rates following contact with chlorhexidine acetate and PHMB were significantly higher than those after contact with povidone-iodine (p<0.05). Nevertheless, the cell survival rates did not significantly differ between the three disinfectants after 180 s of contact time (p>0.05).

#### Cytotoxicity after prolonged direct contact with various disinfectant concentrations

**Figure 2:**
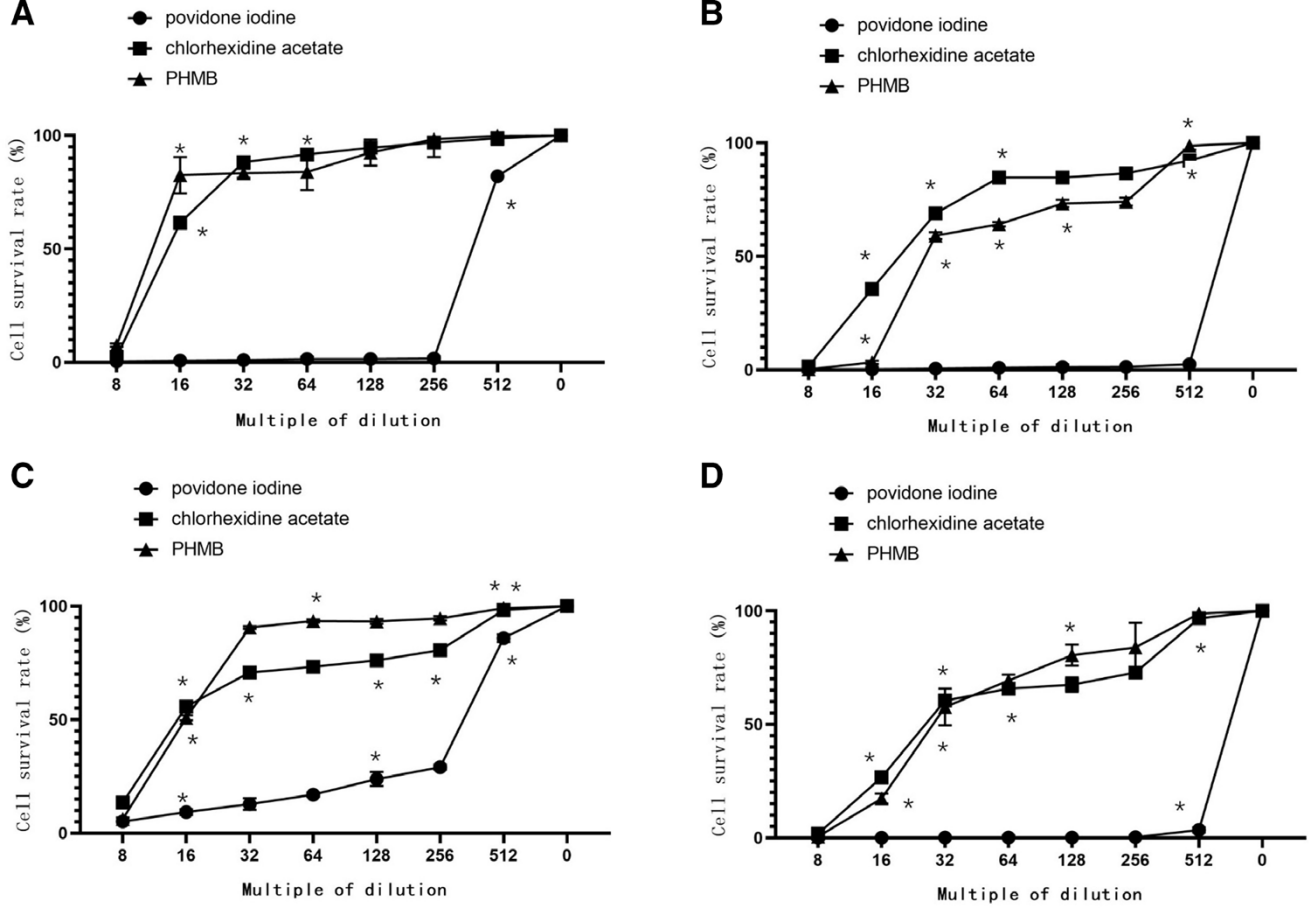
Variation in direct contact cytotoxicity of povidone iodine, chlorhexidine acetate and PHMB with concentration. *Compared with the previous dilution ratio, there was a significant difference (p<0.05). (A, B), HF. (C, D), HaCaT. (A, C) Cytotoxicity after 0.5 hour. (B, D) Cytotoxicity after 4 hour. HF, human fibroblasts; PHMB, polyhexamethylene biguanide.

**Figure 3:**
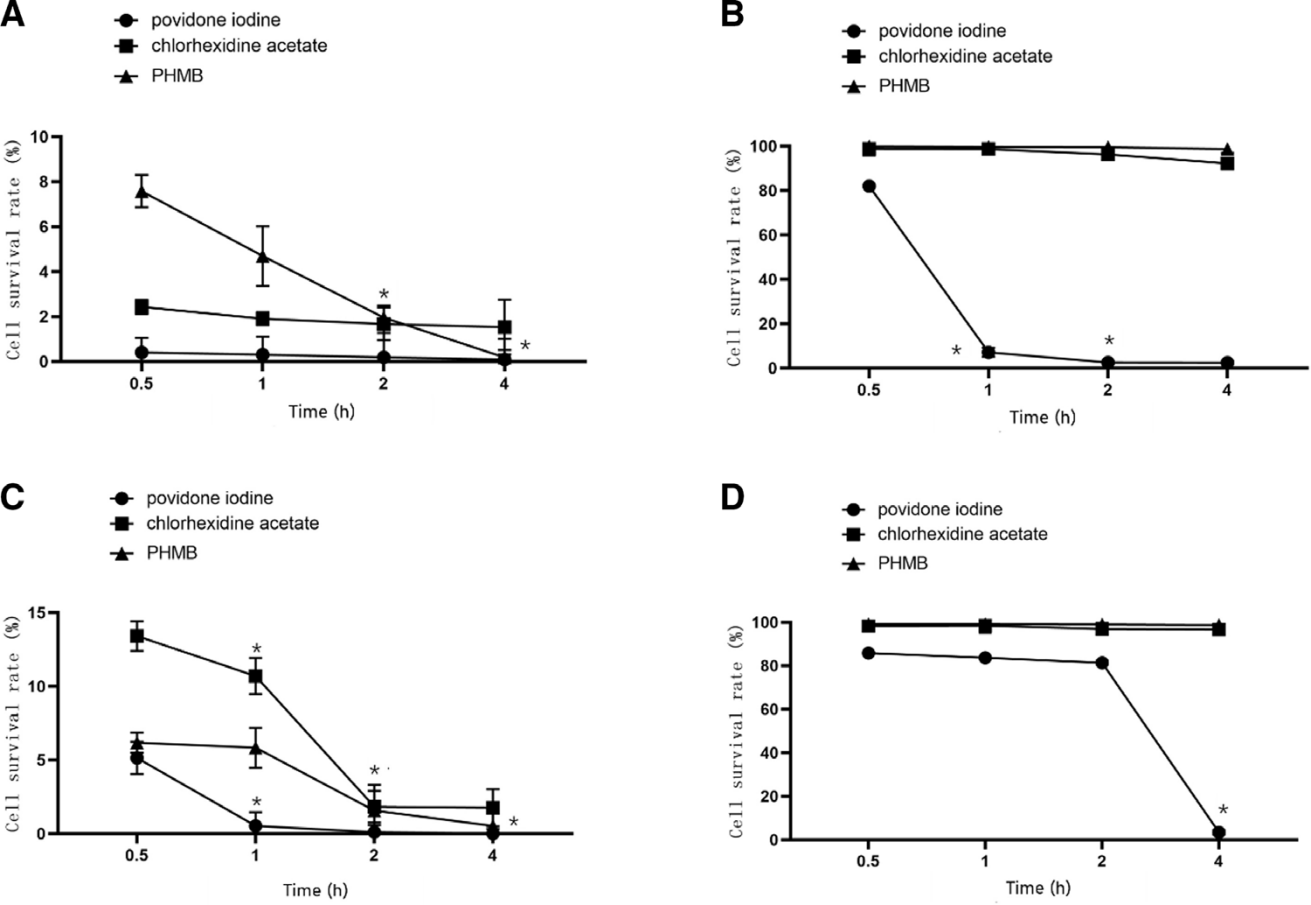
Direct-contact cytotoxicity of various dilutions of povidone iodine, chlorhexidine acetate and PHMB. *Compared with the previous period, there were significant changes, p<0.05. (A, B), HF. (B, C), HaCaT. (A, C) Cytotoxicity of three disinfectants at 1/8 dilution. (B, D) Cytotoxicity of three disinfectants at 1/512 dilution. HF, human fibroblasts; PHMB, polyhexamethylene biguanide.

**Figure 4:**
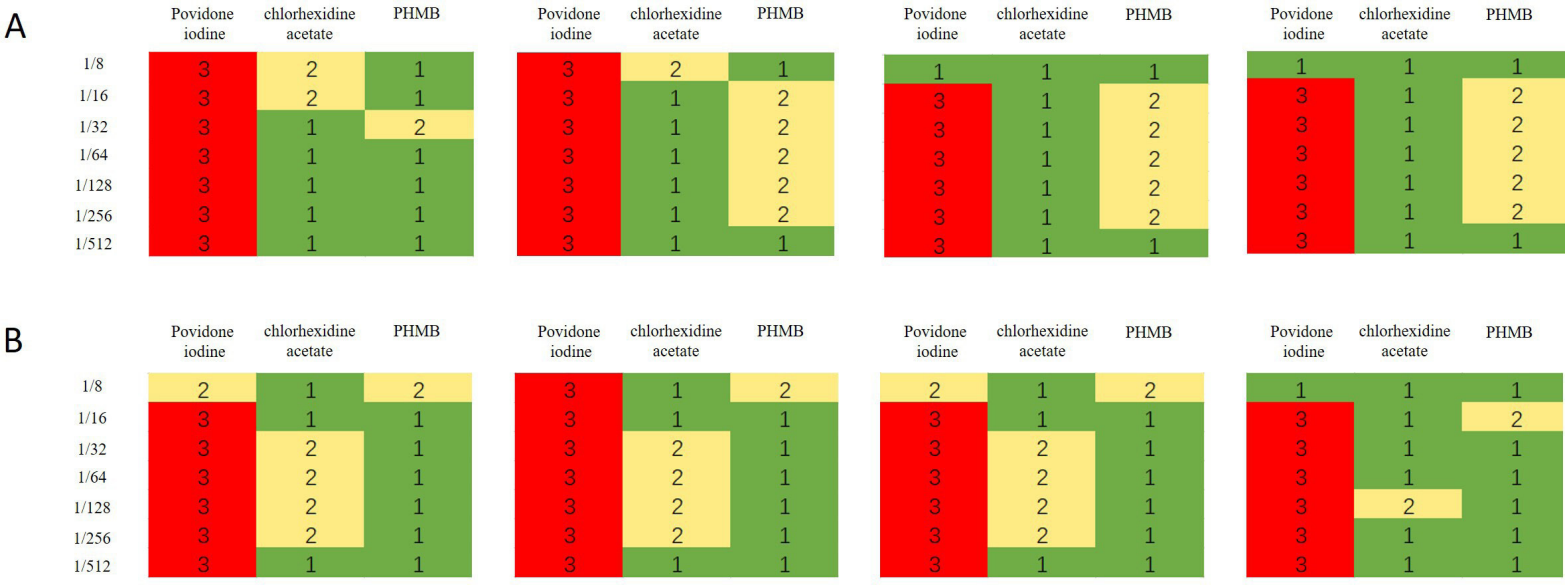
Relative cytotoxicity of povidone iodine, chlorhexidine acetate and PHMB at various dilutions and contact times. For the same contact time and concentration, a <1> or a green background represents the lowest cytotoxicity, a <2> or a yellow background represents intermediate cytotoxicity and a <3> or a red background represents the highest cytotoxicity. Different dilutions with the same number and background colour do not significantly differ in terms of cytotoxicity (p>0.05). (A) Cytotoxicity of the three disinfectants to HF. (B) Cytotoxicity of the three disinfectants to HaCat. HF, human fibroblasts; PHMB, polyhexamethylene biguanide.

Unlike the other disinfectants, povidone-iodine was significantly cytotoxic to both HF and HaCat cells, even at 1/512 dilution (p<0.05). Decreases in povidone-iodine concentrations did not markedly reduce its cytotoxicity to HF. The cytotoxicity of minimal povidone-iodine dilutions was not influenced by contact time either. In contrast, the cytotoxicity of chlorhexidine acetate and PHMB decreased with decreasing concentrations. At 1/512 dilution, PHMB did not significantly affect cell survival rates, regardless of contact time (p>0.05). Furthermore, after a 4-hour exposure to the 1/512 dilution, chlorhexidine acetate significantly lowered the HF survival rate (p<0.05). At minimal dilutions, PHMB cytotoxicity to HF was highly dependent on contact time and the influence of contact time increased with the dilution rate. However, the cytotoxicity of chlorhexidine acetate to HF was less dependent on contact time. At minimal dilutions, the cytotoxicity of chlorhexidine acetate and PHMB on HaCat increased with contact time. However, this correlation decreased with an increase in disinfectant dilution. The results are summarised in figures 2–4 and online supplemental figures S1, S2.

10.1136/bmjnph-2022-000431.supp1 Supplementary data

**Figure 5:**
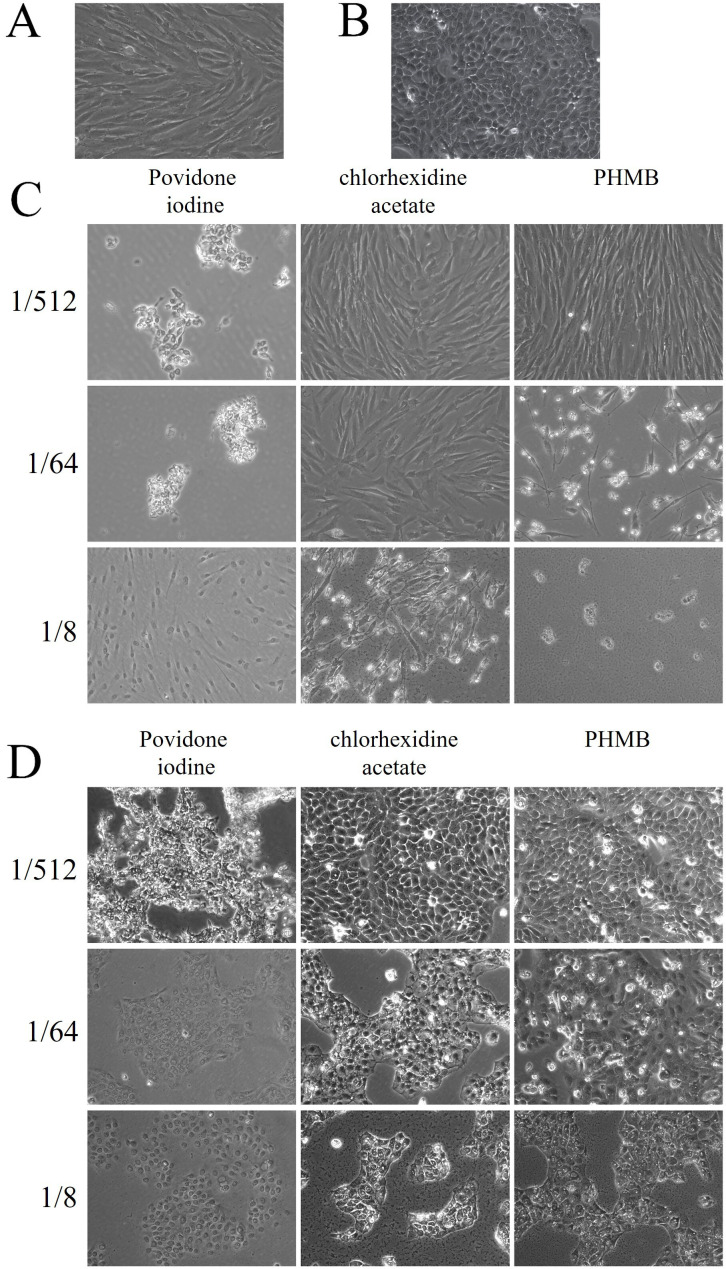
Changes in cell morphology in response to exposure to different dilutions of the three disinfectants. (A) normal HF, (B) normal HaCat, (C) Morphological changes in HF on exposure to the three disinfectants, (D) morphological changes in HaCat on exposure to the three disinfectants after 2 hours. HF, human fibroblasts; PHMB, polyhexamethylene biguanide.

Cell morphology analysis showed that the 1/512 and 1/64 povidone-iodine dilutions destroyed both HF and HaCat, causing their cytoplasms to shrink and exposure of their nuclei. Povidone iodine at a 1/8 dilution had an effect on solidification, similar to paraformaldehyde. The 1/512 dilutions of chlorhexidine acetate and PHMB did not affect the morphology of HF or HaCat cells significantly. However, 1/8 and 1/64 dilutions induced vacuolation in most cells. The results are shown in figure 5.

#### Effects on apoptosis

**Figure 6:**
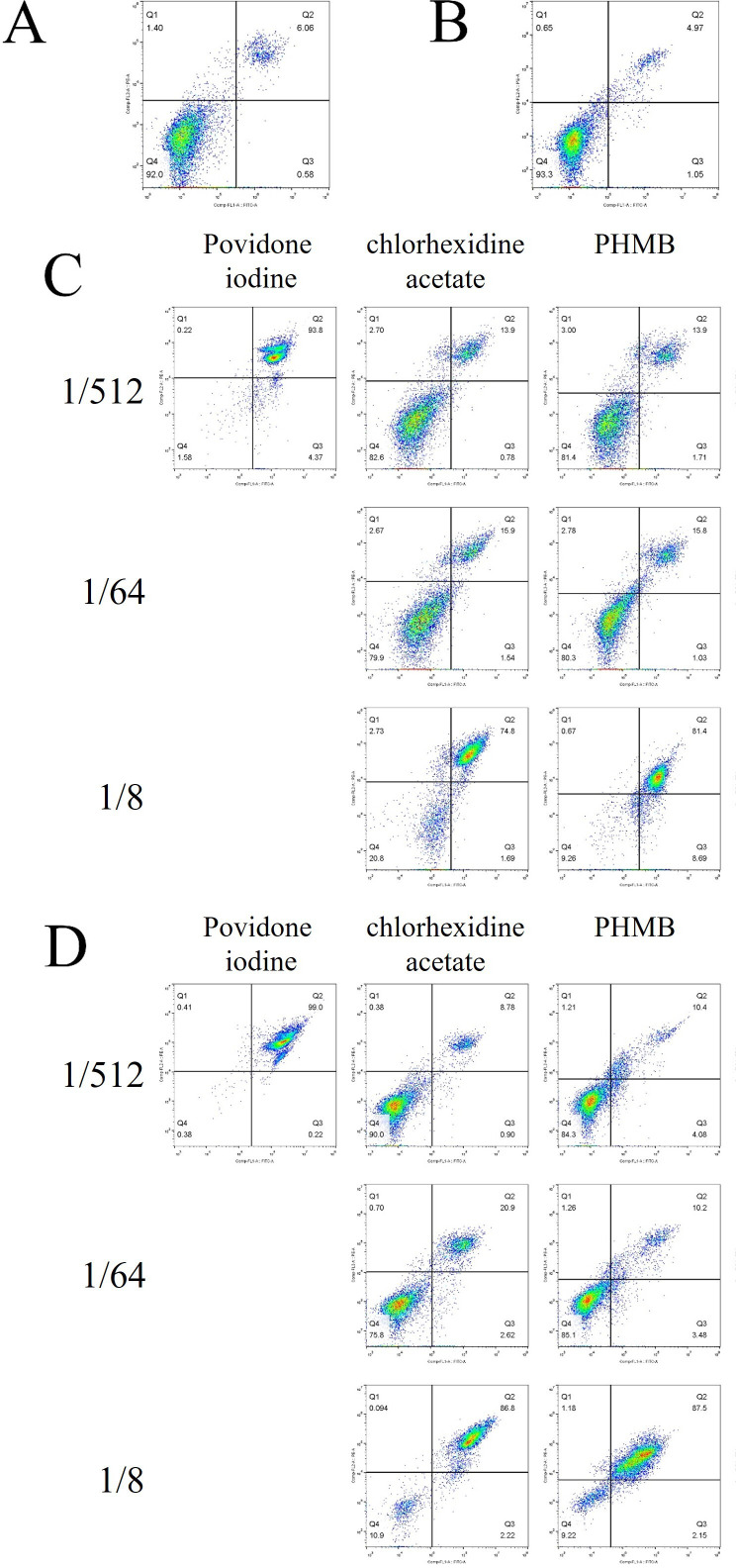
Apoptosis in HF and HaCat. (A) Apoptosis in normal HF cells. (B) Apoptosis in normal HaCat. (C) Apoptosis in HF after contact with disinfectant. (D) Apoptosis in HaCat after contact with disinfectant. Cells treated with 1/8 and 1/64 povidone iodine dilutions were destroyed and could not be detected. HF, human fibroblasts; PHMB, polyhexamethylene biguanide.

Povidone iodine, chlorhexidine acetate and PHMB at 1/8, 1/64 and 1/512 dilutions, respectively, significantly promoted HF and HaCat apoptosis compared with normal, untreated cells (p<0.05). The 1/8 and 1/64 povidone-iodine dilutions fragmented the cells to a point where they were virtually undetectable. The results are summarised in figure 6 and online supplemental table S1.

#### Antibacterial efficacy of disinfectants in vitro

The MIC and MBC values of each disinfectant and the susceptibility rates of the standard strains are listed in table 1. Dilution ratios were used to evaluate the differences in antibacterial efficacy between the disinfectants. Chlorhexidine acetate and PHMB showed similar antibacterial efficacies against *A. baumannii* and *S. aureus*. Both presented superior efficacy against povidone-iodine. PHMB had better antibacterial efficacy against *K. pneumoniae* than either chlorhexidine acetate or povidone-iodine. All three bacteria were more susceptible to PHMB than chlorhexidine acetate. *K. pneumoniae* had greater susceptibility to PHMB than povidone-iodine (p<0.05).

**Table 1:** (A) Apoptosis rate of HF after exposure to three disinfectants at various concentrations (%). (B) Apoptosis rate of HaCat after exposure to three disinfectants at various concentrations (%)

	Povidone iodine		Chlorhexidine acetate		PHMB	
	Early apoptosis	Late apoptosis	Early apoptosis	Late apoptosis	Early apoptosis	Late apoptosis
(A)						
1/512	4.34±0.36	93.83±1.86	0.78±0.57	13.93±2.06	1.71±0.73	13.90±2.98
1/64	–	–	1.54±0.36	15.93±2.96	1.03±0.32	15.83±2.12
1/8	–	–	1.69±0.23	74.80±2.56*	8.69±2.05*	81.43±3.39*
(B)						
1/512	0.16±0.07	99.43±0.35	0.96±0.35	8.78±0.17	4.08±0.45	10.39±1.06
1/64	–	–	2.62±0.89*	20.90±2.15*	3.48±0.54	20.17±5.70*
1/8	–	–	2.22±0.20	86.77±5.49*	2.15±0.16	87.50±3.17*

*Statistically significant difference compared with previous dilution; p<0.05.

–, unable to detect; HF, human fibroblasts; PHMB, polyhexamethylene biguanide.

## Discussion

For burns,^14^ trauma wounds and diabetic feet,^15^ the application of topical antimicrobials is vital when there is suspected risk or frank evidence of wound infection. When selecting an appropriate disinfectant, both its antibacterial efficacy and toxicity to wound cells must be considered.

Here, we selected and compared povidone-iodine, chlorhexidine acetate and PHMB, which are all commonly used in clinical therapy and have broad-spectrum bactericidal efficacies.^10^ In this study, we set disinfectant concentrations that are frequently used in clinical practice. Dilution ratios were used to identify cytotoxicity and bactericidal efficacy. In this manner, horizontal comparisons can be made and misinterpretations often associated with comparing absolute concentrations can be avoided.^16^ The bacteria tested are species typically found in wounds and the two cell types, HF and HaCat, used in the experiments, are known to play important roles in wound healing.

Studies have shown that the direct application of standard disinfectants to wounds significantly reduces cellular activity, even when the contact time is only 5 s. This could impair or delay wound healing. However, the wound is complete, and there is some compensation. Moreover, the presence of dead cells on the wound surface may impede access of the disinfectant to wound cells.^17^ This suggests that it is more beneficial to apply normal saline than disinfectants to aid wound healing in cases where there is no evidence of obvious wound infection.

Disinfectants are not immediately eliminated on contact with the wound. Certain slow-release disinfectant formulations maintain prolonged contact with the wound tissue. The residual concentration of slow-release disinfectants was relatively low. Nevertheless, our study suggested that, even at very low disinfectant concentrations, prolonged exposure may cause substantial cytotoxicity. Povidone-iodine was the most cytotoxic of the three disinfectants tested in this study, with a significant impact on cellular activity. Even when diluted to 1/512 of the standard concentration, povidone-iodine induced pronounced morphological changes in cells. At higher concentrations, povidone-iodine solidified the cells. In contrast, chlorhexidine acetate and PHMB had relatively lower cytotoxicity than povidone-iodine. In general, cytotoxicity decreased with contact time and concentration. At a 1/512 dilution, neither chlorhexidine acetate nor PHMB had a dramatic effect on cell survival. However, chlorhexidine acetate was less toxic to HF, whereas PHMB was comparatively less toxic to HaCat. Thus, PHMB is suitable for the treatment of shallow wounds primarily repaired by keratinocyte growth and migration.

All three disinfectants showed similar effects on apoptosis. Therefore, disinfectants may have negative impacts on the overall long-term wound healing prognosis as they increase apoptosis and are directly cytotoxic.

*S. aureus*, *A. baumannii* and *K. pneumoniae* are the common causative bacteria in wound infections. Povidone-iodine, chlorhexidine acetate and PHMB have good antibacterial efficacy against these species. The MICs and MBCs of all three disinfectants were significantly lower than the standard concentrations. Nevertheless, the MIC and MBC dilution ratios for povidone-iodine were lower than those for the other two disinfectants. Thus, povidone-iodine has a relatively weak antibacterial efficacy in vitro.

Despite the useful data reported, this study had certain limitations. Cytotoxicity tests were conducted in vitro. This environment is physically different from that of wounds. Our research raises some questions regarding traditional wound treatment. If there is no risk of infection in the wound, it is not necessary to use a disinfectant. However, determining whether there is a risk of infection in the wound is a new and difficult task.

## Conclusion

In vitro analyses of the cytotoxicity and antibacterial efficacy of povidone-iodine, chlorhexidine acetate and PHMB and offered a new perspective on topical disinfectant application for wound treatment and healing. All three disinfectants were cytotoxic, with povidone-iodine being the most cytotoxic even at low concentrations. PHMB had better antibacterial efficacy against *S. aureus* and is suitable for the treatment of shallow wounds primarily. All three tested bacteria were significantly more sensitive to PHMB than to the other disinfectants. It may be more beneficial to apply normal saline only to aid wound healing; however, in cases where there is evidence of obvious wound infection it is necessary to pay attention to the use time and concentration of the disinfectant to prevent cellular damage.

## Data Availability

All data relevant to the study are included in the article or uploaded as online supplemental information. Data can be accessed through jinjiannavy@163.com.
