# Supplementary material for: Analysis of povidone iodine, chlorhexidine acetate and polyhexamethylene biguanide as wound disinfectants: in vitro cytotoxicity and antibacterial activity
Source: BMJ Nutr Prev Health. 2023 Apr 4;6(1):21–7. doi: 10.1136/bmjnph-2022-000431 (PMC10407393; doi:10.1136/bmjnph-2022-000431)
Supplement: Supplementary data [file bmjnph-2022-000431supp001.pdf]

Table S1-1a. Apoptosis rate of HF after exposure to three disinfectants at various concentrations (%)

	Povidone iodine		Chlorhexidine acetate		PHMB	
	Early apoptosis	Late apoptosis	Early apoptosis	Late apoptosis	Early apoptosis	Late apoptosis
1/512	4.34 ± 0.36	93.83 ± 1.86	0.78 ± 0.57	13.93 ± 2.06	1.71 ± 0.73	13.90 ± 2.98
1/64	-	-	1.54 ± 0.36	15.93 ± 2.96	1.03 ± 0.32	15.83 ± 2.12
1/8	-	-	1.69 ± 0.23	74.80 ± 2.56*	8.69 ± 2.05*	81.43 ± 3.39*

-unable to detect; *statistically significant difference compared with previous dilution; $P < 0.05$

Table S1-1b. Apoptosis rate of HaCat after exposure to three disinfectants at various concentrations (%)

	Povidone iodine		Chlorhexidine acetate		PHMB	
	Early apoptosis	Late apoptosis	Early apoptosis	Late apoptosis	Early apoptosis	Late apoptosis
1/512	0.16 ± 0.07	99.43 ± 0.35	0.96 ± 0.35	8.78 ± 0.17	4.08 ± 0.45	10.39 ± 1.06
1/64	-	-	2.62 ± 0.89*	20.90 ± 2.15*	3.48 ± 0.54	20.17 ± 5.70*
1/8	-	-	2.22 ± 0.20	86.77 ± 5.49*	2.15 ± 0.16	87.50 ± 3.17*

-unable to detect; *statistically significant difference compared with previous dilution; $P < 0.05$

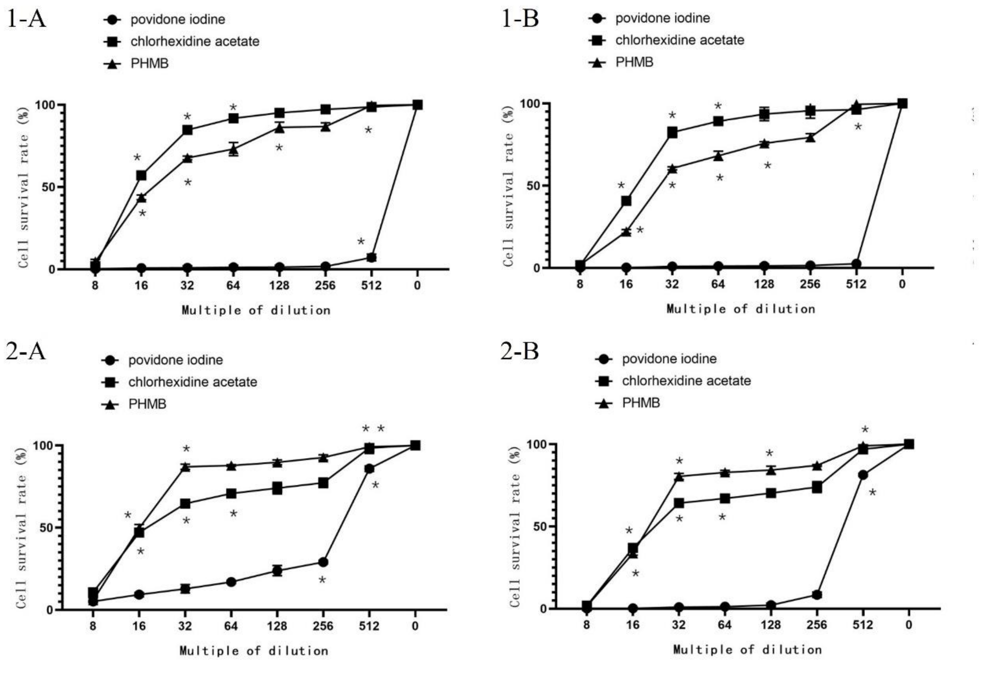

Figure S1. Variation in direct contact cytotoxicity of povidone iodine, chlorhexidine acetate, and PHMB with concentration . *Compared with the previous dilution ratio, there was a significant difference ($P < 0.05$). Figure S1-1: HF. Figure S1-2: HaCaT. **A:** Cytotoxicity after 1 hour. **B:** Cytotoxicity after 2 hour.

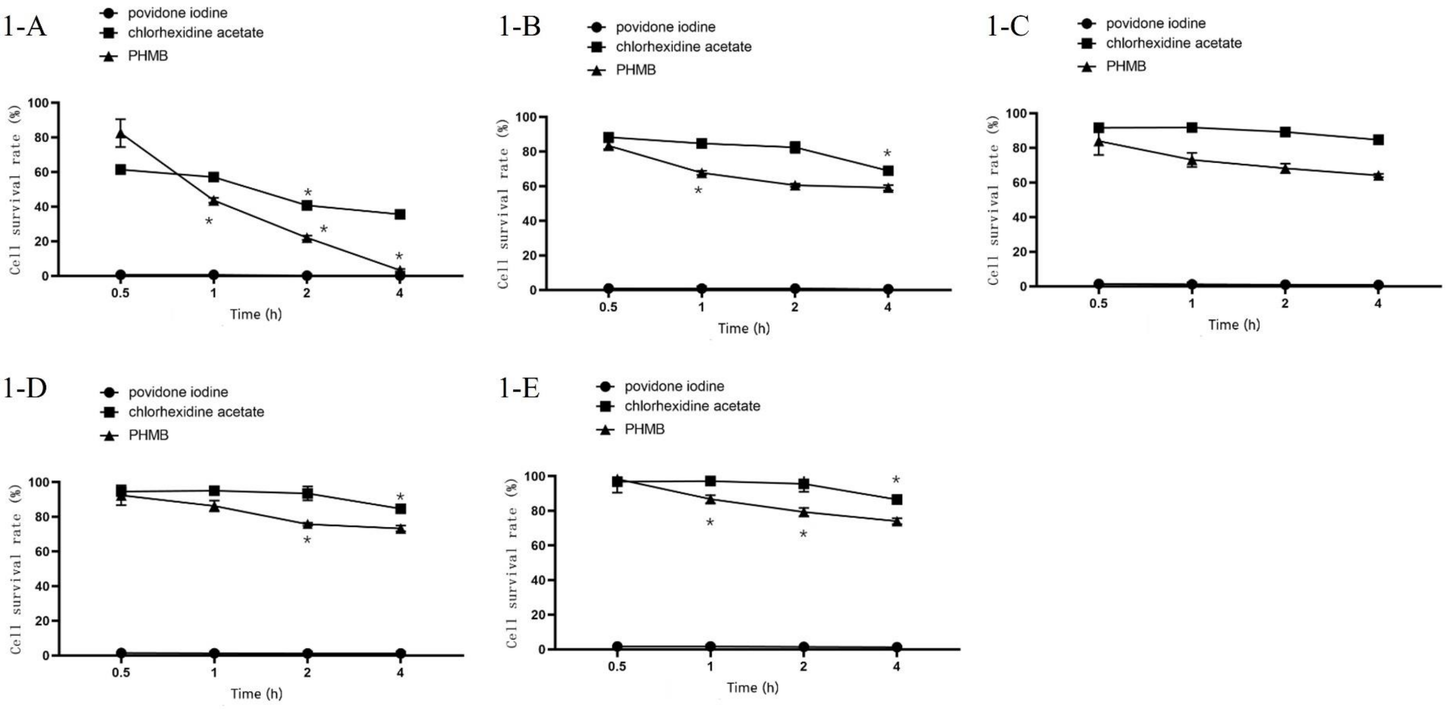
1-A
1-B
1-C
1-D
1-E
povidone iodine
chlorhexidine acetate
PHMB
Cell survival rate (%)
Time (h)
0.5
1
2
4
0
20
40
60
80
100
*

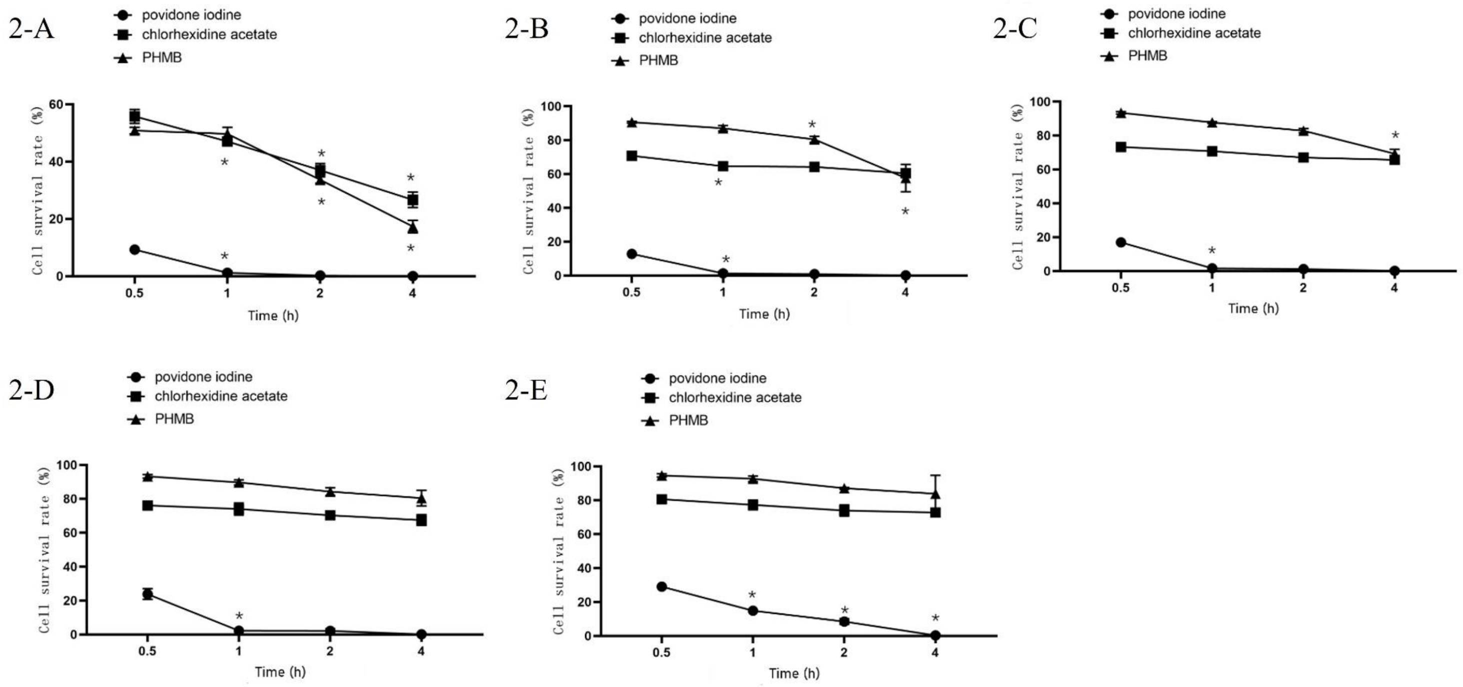

Figure S2. Direct-contact cytotoxicity of various dilutions of povidone iodine, chlorhexidine acetate, and PHMB. *Compared with the previous period, there were significant changes, $P < 0.05$. Figure S2-1: HF. Figure S2-2: HaCaT. **A:** Cytotoxicity of three disinfectants at 1/16 dilution. **B:** Cytotoxicity of three disinfectants at 1/32 dilution. **C:** Cytotoxicity of three disinfectants at 1/64 dilution. **D:** Cytotoxicity of three disinfectants at 1/128 dilution. **E:** Cytotoxicity of three disinfectants at 1/256 dilution.
